# Cloud point extraction coupled with back extraction: a green methodology in analytical chemistry

**DOI:** 10.1080/20961790.2019.1643567

**Published:** 2019-09-18

**Authors:** Shivpoojan Kori

**Affiliations:** Chemistry, Biochemistry & Forensic Science, Amity School of Applied Sciences, Amity University Gurgaon (Manesar), Haryana, India

**Keywords:** Forensic sciences, forensic toxicology, surfactant, cloud point extraction, alkaloid, drug, organophosphorus compound

## Abstract

Recently, cloud point extraction (CPE) coupled with back extraction (BE) has been suggested as a promising alternative to liquid-liquid extraction. In CPE, non-ionic surfactants in aqueous solutions form micelles and the solution becomes turbid when heated to the cloud point temperature. Microwave- or ultrasonic-assisted BE can be performed after CPE and before injection of the sample for instrumental analysis by ultraviolet-visible spectroscopy, high-performance liquid chromatography, gas chromatography, gas chromatography-mass spectrometry, or liquid chromatography-mass spectrometry. This article reviews selected published scientific research on the application of CPE-BE to the determination of alkaloids, drugs and organophosphorus compounds from several complex matrices. This method could be scaled-up for use in forensic science.

## Introduction

In forensics, novel surfactant chemistry using surfactant aggregates can be applied to extract various polar and non-polar components [1]. Within the framework of detection and identification, extraction of the analyte in question is of paramount importance. The efficiency of extraction depends upon a number of factors, including analytical requirements and the nature of solvents and toxicants [2]. The sample matrix is also important, and matrices can be solids (e.g. soil, plant tissue, vegetable, fruit and tablet samples), semi-solids (e.g. cream, gel, suspension and colloid samples), or liquids (e.g. serum, plasma, whole blood, milk, water and fruit juice samples). The physicochemical composition of the sample matrix from which the target analyte is extracted affects the extraction efficiency. Consequently, sample preparation can play a key role in identification and quantification of target analytes. It is well-known that sample preparation has a direct impact on accuracy, precision and quantification limits. The principle motivations behind sample pre-treatment are: (i) extraction of the target analyte from the matrix, (ii) removal of proteins and other compounds that could interfere with the analysis, and (iii) modification of the pH, ionic strength and concentration of the sample to optimize the extraction. For most matrices, some form of sample pre-treatment (e.g. dilution) is required before analysis. The most frequently used pre-treatment techniques are protein precipitation, liquid-liquid extraction and solid-phase extraction. For instance, lipophilic drugs are associated with fats and glycerol molecules in biological samples. Analysis of highly hydrophilic molecules in biological matrices creates challenges because of low recovery of analytes. However, it is very difficult to separate these drugs from these complex biological matrices. Consequently, a highly selective and sensitive methodology is required for sample preparation.

The extraction and identification of drugs of abuse in biological matrices, such as plasma, whole blood and serum, is a common requirement in forensic laboratories [3]. In toxicological examinations, a coordinated search is performed first with a constrained number of substances. Then, an undirected inquiry is performed in a systematic toxicological examination, which looks for possibly toxic substances whose presence is suspicious and whose quantities are unknown. Systematic toxicological examination is required if almost no data are available in cases involving an unknown. The drug screening process can be generally categorized into two phases: (i) sample preparation and (ii) analysis of the drugs. The samples available for investigation are typically complicated biological matrices, in which the toxicological substances of interest are present in trace amounts compared with endogenous compounds. The extraction of analytes such as alkaloids, drugs and organophosphorus (OP) compounds from biological matrices is a challenging and time-consuming task in forensic science laboratories [4]. Therefore, it is imperative that work-up procedures retain as much as possible of the target substance and remove other substances and potential interferences. An array of surfactant assemblies in aqueous or non-aqueous media can be used for efficient recovery of target analytes in such cases.

## General considerations

### Surfactants

A surfactant is a material that, when present in a low concentration, adsorbs onto an interface or surface and alters the interface free energy [5]. In other words, surfactants display interfacial associations by means of enhanced assimilation at the interface. Surfactants are amphiphilic molecules with both hydrophobic and hydrophilic components and are important in chemical technology.

**Figure 1.**
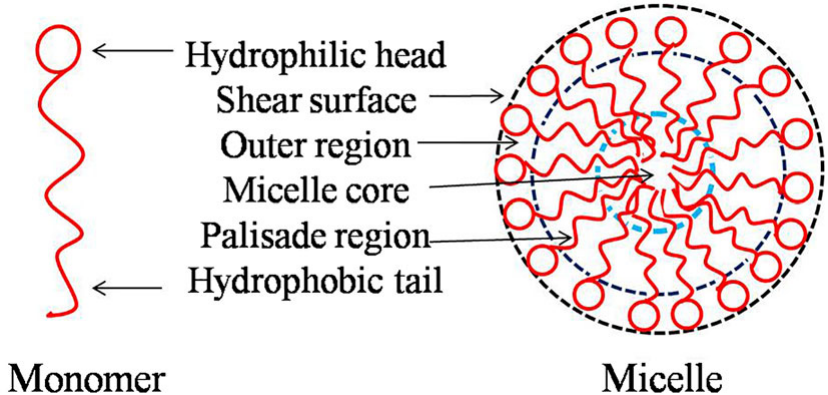
Locus of solubilization of an analyte in a micelle.

Surfactants have characteristic chemical structures with a part that has affinity for the bulk solvent and a part that has a strong affinity for a hydrophilic or lipophilic group. The hydrophobic group or tail of the surfactant contains at least one hydrocarbon chain of 6–20 carbon atoms. The tail can be branched or linear; aliphatic, alkyl or aryl; and short or long. The surfactant head group can be ionic or non-ionic. When a low concentration of a surfactant is dispersed in an aqueous solution, the surfactant is generally found in a monomeric or dimeric state. When the surfactant concentration is increased above a certain threshold, called the critical micellar concentration (CMC), these monomers or dimers spontaneously aggregate to form colloidal-sized clusters known as micelles. When dispersed in water, the micelles have a hydrophilic surface and hydrophobic core. This structure means the micelles can interact chemically or physically with either hydrophilic or lipophilic analytes to enhance their solubilities. This makes surfactants excellent vectors for extraction and isolation of drugs and polyphenolic compounds [6]. For solubilization of an analyte in a micellar system, the maximum quantity of analyte that can be incorporated into a given surfactant formulation is termed the maximum additive concentration. Solubility data are expressed in a solubility versus concentration curve or a three-component phase diagram, which describes the effects of varying all three components of the system (i.e. analyte, surfactant and solvent). The site of solubilization within the micelle is closely related to the chemical nature of the analyte (Figure 1).

Surfactants can be categorized depending on the nature of their hydrophilic head group [5]. An anionic surfactant has a negatively charged moiety as its polar head group, such as a sodium alkylsulfate (e.g. sodium dodecyl sulfate, sodium decyl sulfate or sodium tetradecyl sulfate) or sodium alkylcarboxylate. Cationic surfactants have a positively charged head group such as an alkyl pyridinium halide or alkyl ammonium halide (e.g. hexadecyl (or cetyl) trimethylammonium bromide or cetyltrimethylammonium chloride) or a quaternary ammonium salt (e.g. hexadecyl pyridinium bromide, carbethopendecinium bromide (Septonex)). Non-ionic surfactants generally have a polar, uncharged head group, and include surfactants such as polyoxyethylene glycol octylphenol ethers or polyoxyethylene glycol tert-octylphenol ethers, which vary in their number of ethylene oxide repeating units. Some well-known surfactants are the Triton, Tween and Brij series. Finally, zwitterionic (amphoteric) surfactants have both a cationic and anionic polar head group (e.g. alkyl ammonium ethyl sulfates, phosphobetaines, lecithins or sulfobetaines).

The degree of hydrophilicity or lipophilicity of a surfactant can be decided according to Griffin’s arbitrary scale [7,8], which is derived from the ratio of hydrophilic-hydrophobic character. Because of its amphiphilic nature, a surfactant can function as a solubilizer, wetting agent, emulsifier, and permeability enhancer. Its adsorption behaviour depends upon the solvent and chemical structure of the surfactant. Surfactants are particularly varied in terms of their nature and physical properties, ability to radically alter surface and interfacial properties, and tendency to self-associate and solubilize themselves in micelles (Table 1). Because of these properties, surfactants are useful for the solubilization and extraction of analytes [9].

**Table 1.** Physiochemical characteristics of the surfactants.

Surfactants	Avg mol wt.	CMC (mmol/L)	CP (°C)	HLB	Agg.#	Reference
SDS (C_12_H_25_ONaO_4_S)	288.4	8	>100	40	60	[10]
Tween 80 (C_64_H_124_O_26_)	1 310	0.015	65	15	60	[11]
Span 40 (C_22_H_42_O_6_)	402.57	–	–	6.7	–	[9]
Brij-35 (C_58_H_118_O_24_)	1 225	0.09	>100	16.9	40	[12]
Brij-58 (C_56_H_114_O_21_)	1 122	0.077	>100	15.7	70	[13]
Triton X-45 (C_8_H_17_C_6_H_4_O(CH_2_CH_2_O)_5_H)	427	136^a^	Dispersible	9.8	140	[12]
Triton X-100 (C_8_H_17_C_6_H_4_(OC_2_H_4_)_10_OH)	∼625	0.2–0.9	65	13.5	65	[12]
Triton X-114 (C_8_H_17_C_6_H_4_O(CH_2_CH_2_O)_7.5_H)	537	∼0.2	25	12.4	60	[12]

Avg mol wt.: average molecular weight; CMC: critical micellar concentration; CP: cloud point; HLB: hydrophilic–lipophilic balance; Agg.#: aggregation number.

^a^ppm.

### Analytes of forensic interest

#### Alkaloids

Many plants produce alkaloids that can have deleterious effects on human body and can even lead to death [14]. Poisoning with these compounds via ingestion of the whole or part of a plant is chiefly classified into one of three major categories: (i) accidental, (ii) intentional and (iii) abuse. Plant poisoning is frequently encountered in homicide and suicide cases and has been identified as the cause of death in many cases. Toxic plant use is encountered in criminal cases, and alkaloids from whole plants and their parts have been abused for their psychoactive effects [15]. Toxicological investigations of these alkaloids could aid in identification of poisoning or abuse cases. From a forensic perspective, it is imperative that accurate methods are available for unambiguously identifying these alkaloids [16]. These methods can also be used for extraction of beneficial plant extracts.

#### Drugs

Illicit drug use has an immense social impact. At the individual level, drug abuse has been connected to psychological disorders, violent behaviour and involvement in criminal activity and traffic accidents. Drug abuse is strongly correlated with accidental injuries from traffic accidents, drowning, poisoning, burns and pre-meditated injuries. Recently, new psychoactive compounds that are neither officially registered for therapeutic use nor scheduled as controlled substances, at least not when they first appear on the recreational/illicit drug market, have become popular. These compounds are often sold as so-called “legal highs” and/or labelled as harmless products, such as incense, bath salts or plant food. Testing for drugs in biological samples can provide important information on drug use or abuse by individuals and is a key task in forensic toxicology and allied fields. Testing can confirm a suspected acute drug influence or intoxication/poisoning (e.g. driving under the influence of drugs or postmortem toxicology). It can also be used for monitoring abstinence from drug abuse, for example, in workplace drug testing, drug withdrawal or substitution treatment, or a drug-abstinence programme for re-granting of a driver’s license.

#### OP compounds

OP compounds are widely utilized in pest prevention and treatment. They have a number of advantages but also disadvantages, for example, their potential toxicity to humans and other animals. Because of their potential toxicity, OP compounds are often encountered as toxins in homicide and suicide cases. In forensic laboratories, the suspect poisoned food or beverage is analysed to determine the presence of any potentially toxic substances in the sample. These investigations can confirm the connection between a toxic substance and cause of death. This is of utmost significance and demonstrates the relationship between toxicology and forensic medicine, which is facilitated by highly sensitive techniques and specific extraction protocols. The wide array of matrices and OP compounds that can be encountered in criminal cases requires the development of detection and quantification strategies with adequate precision and accuracy for OP residues in a variety of samples (e.g. foods, beverages and biological matrices).

### Surfactant systems compared with conventional solvents

Liquid–liquid separation has been used for non-ionic or zwitterionic surfactant micelles but has been limited for charged surfactant species. Surfactant-mediated extraction has the following advantages:

- Surfactants have characteristic properties that make them promising extraction vectors.
- The small volume of the surfactant-rich phase obtained with this methodology permits the design of extraction schemes that are simple, cheap, highly efficient, user friendly, do not use flammable solvents, result in no loss of analyte during the evaporation of solvents and avoid absorption of non-polar analytes [17–19].
- The operating conditions applied in cloud point extraction (CPE) techniques allow for pre-concentration of thermally sensitive analytes, such as molecules of biological and environmental interest. The pre-concentration factors are comparable or superior to those of other methods, and can be adjusted by varying the amount of surfactant.
- CPE coupled with back extraction (BE) can be used to extract and pre-concentrate analytes from complex matrices [2,20].
- Surfactant-mediated extraction and pre-concentration is a green chemistry technique.

### Principles of surfactant-mediated extraction and CPE

**Figure 2.**
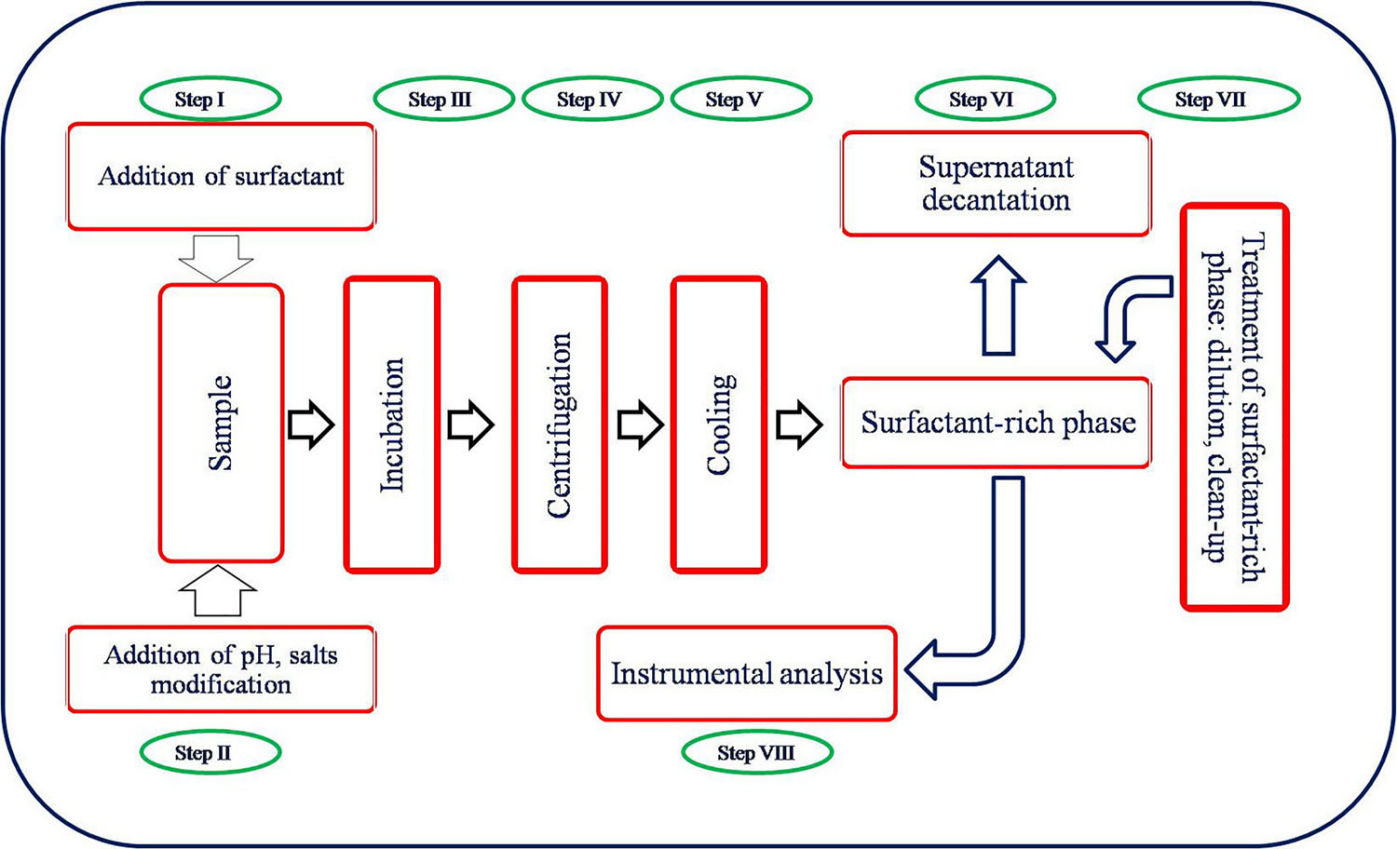
Schematic diagram of the cloud point extraction system for analytes from complex matrices.

In CPE, the role of extraction solvent is played by the micellar (surfactant-rich) phase originating from a homogenous surfactant solution that is added to the sample. In aqueous dispersion media, the surfactant aggregate orientates its hydrocarbon tail towards the centre to create a non-polar core. Hydrophobic compounds, which include many bioactive compounds, present in the aqueous solution are isolated and partitioned in the hydrophobic core of the micelles [21–23]. With a decrease in the number of polyethoxylate groups (Ethylene Oxide number) or an increase in the alkyl carbon number, intermicellar attractive forces increase and the cloud point decreases [24]. CPE consists of eight simple steps: (i) addition of surfactant; (ii) pH maintenance for solubilization of the analytes in the micellar aggregates by providing favourable pH environment; (iii) incubation for clouding; (iv) centrifugation; (v) cooling; (vi) phase separation for analysis; (vii) pre-treatment of the surfactant-rich phase and (viii) instrumental analysis (Figure 2). In the aqueous solution, the unique structure of the surfactant allows for sparingly soluble or water-insoluble substances to be solubilized because they can associate and bind to the micellar assembly. The interactions between the surfactant and analyte may be electrostatic, hydrophobic or a combination of both. When a surfactant solution is heated over a critical temperature, the solution easily separates into two distinct phases: one contains the surfactant at a concentration below or equal to the CMC, and the other is a surfactant-rich phase. The hydrophobic compounds initially present in the solution and bound to the micelles are extracted into the surfactant-rich phase. This phenomenon is particularly obvious for polyoxyethylene surfactants and can be attributed to the two ethylene oxide segments in the micelle, which repel each other at low temperature when they are hydrated and attract each other when the temperature increases because of dehydration. The theory and relevant applications of this impressive separation method are discussed in various reviews [18,25–27]. CPE mainly depends on solubilization of the surfactant solution and phase separation for the extraction and pre-concentration of analytes. The use of micellar systems as an alternative to other separation techniques offers several advantages, including low cost, safety and high capacity to concentrate a wide range of analytes with widely varying natures with high recoveries and very high concentration factors. The extraction efficiency for the target analyte by CPE is influenced by many factors, such as the pH of the sample, solution, surfactant type and concentration, temperature and time to reach equilibrium and ionic strength. The effects of these factors on the extraction of analytes need to be established.

### Influencing parameters

There are many factors that affect CPE efficiency, such as the type of surfactant, surfactant concentration, pH, ionic strength, centrifugation, incubation and saturation and the Krafft temperature. When developing a CPE method, several parameters need to be taken into account.

#### Type of surfactant

The solubilization/partitioning of non-polar organic molecules in a hydrophobic micellar core is an inherent property of all surfactant systems. The efficiency of this step is dependent on the magnitude of analyte solubilization in the micelle (i.e. non-polar core and polar micelle-water interface), analyte polarity and solution composition. Several non-ionic surfactants (e.g. TX-100 (polyoxyethylene(9.5)-*t*-octylphenol), Brij-97, PONPE 7.5) have been tested and compared as extractants for analytes. For TX-100, significant spectral interference was observed in the analysis of terazosin hydrochloride because of peak overlap between the analyte and surfactant [28]. For Brij-97, the extraction was non-quantitative and a high temperature was necessary to produce clouding [28]. Generally, extraction is more efficient if phase separation is easy. When a highly hydrophobic surfactant is used, the extraction efficiency will be higher and analytical signal will be easier to detect than with a less hydrophobic surfactant. A number of non-ionic surfactants such as Triton X-45, Triton X-100, Triton X-114, Tween 20, Tween 80 and Genapol X-080 have been trialled as extraction solvents [29], and the Triton series was found to be superior to the Tween series. Triton X-114 and Triton X-45 have convenient CPE temperatures but the CPE temperatures for other surfactants, such as Triton X-100 and Tween-80, are too high [30,31]. The peak for Triton X-114 in the chromatogram was small, whereas that for Triton X-45 was large. Triton X-100 gave higher coacervate phase volumes, which made the BE process difficult. To achieve quantitative extraction of the analytes in the BE, it is necessary to minimize the coacervate phase volume to avoid forming a stable emulsion. Triton X-114 has been used in many studies [32–34]. Genapol X-080 is a polyoxyethylene glycol mono ether-type surfactant that contains eight oxyethylene units and tridecyl alkyl moieties [HO(CH_2_CH_2_O)_8_(CH_2_)13H]. It has a molecular weight of 553, CMC of 0.05 mmol/L (0.028 g/L), ratio of hydrophilic-hydrophobic character of 13, and cloud point temperature of 42 °C in pure water [35,36]. Because it possesses no aromatic moiety, Genapol X-080 does not absorb above 210 nm and it does not interfere with the determination of analytes such as atrazine, daidzein, aesculin and aesculetin [35–37]. Tergitol 15-S-7 has been used for extraction of polycyclic aromatic hydrocarbons (PAHs) because it does not interfere with their fluorescence detection [25]. Finally, I conclude that non-ionic surfactants are far better than conventional solvents (such as ethanol, methanol, hexane, benzene, etc.) for extraction purpose.

#### Effect of the surfactant concentration

Above the optimum surfactant concentration, the analytical signal deteriorates because of the increase in surfactant volume; however, if the surfactant concentration is decreased below the recommended level, the accuracy and reproducibility of the analysis suffer [30]. As the temperature increases, dehydration decreases the volume of the surfactant-rich phase. Studies have indicated that CPE should be performed at a temperature higher than the cloud point for the two phases (i.e. water-micelle phase and micelle-rich phase) to be maintained. However, at low surfactant concentrations, the surfactant-rich phase is not sufficient for reproducible extraction and separation [37–39]. The Genapol X-080 concentration has been studied in the range of 0.4%–2.0% (w/v). When the concentration of surfactant was below 1.2%, it was suspended in the bulk solution and difficult to separate into two phases. When the surfactant concentration was increased to 2.0%, although the extraction recovery of analytes increased, the solution becomes too viscous to handle. According to the experimental results, selection of the surfactant concentration is very important for maximizing the extraction recovery [37]. Optimization of the surfactant concentration is important for obtaining the best analytical signals and highest extraction efficiencies [40].

#### Effect of the pH

In CPE, the pH of the sample solution is a critical factor that controls the degree of partitioning of the analytes in the surfactant-rich phase. In ionizable species, the maximum extraction efficiency is achieved at pH values where the non-ionized form of the analytes exists. The pH plays an important role in improving the extraction efficiency in CPE of metals without the addition of a chelating agent. It affects the overall charges of the analytes, and this affects the formation of a complex between the analyte and the surfactant [41]. The role of pH is inline with that in traditional pH-selective fractional precipitation, where the separation of several analytes is made possible by adjusting the pH [16,18]. In the case of ionizable organic analytes, partitioning of the analytes in two immiscible phases depends on the solution pH. The pH affects the extraction of analytes through the formation of ion pairs between the analytes and surfactant assemblies [37]. Maximum extraction efficiency is achieved at pH values where the non-ionized form of the target analytes prevails [42].

#### Effect of ionic strength

The addition of salt (e.g. NaCl, KCl, Na_2_SO_4_, Na_3_PO_4_, KNO_3,_ CaCl_2_ or NaClO_4_) to the solution may influence the extraction process. For most non-ionic surfactants, the presence of a salt may facilitate phase separation because it increases the density of the aqueous phase. Two types of electrolytes are added to formulations because the cation (i.e. Na^+^) may decrease the cloud point from dehydration of the polyoxyethylene chain and the anions (i.e. Cl^−^ and SO_4_^2−^) are likely to decrease self-association of water molecules [43]. Electrolytes fulfil two important purposes: assisting demulsification and decreasing the cloud point temperature. When the mass is less than the optimum amount, the effect of demulsification is incomplete, which means the final surfactant-rich phase contains many impurities. Addition of a quantity of Na_2_SO_4_ above the optimum may result in coagulation and formation of a precipitate, which would make the solution difficult to dilute to scale. The most frequently used salts are Na_2_SO_4_ and NaCl [29]. However, the addition of salts depends on the type and nature of analytes and surfactants [42]. Increasing the ionic strength enhances phase separation through salting out phenomena that also apply to conventional extractions, yielding higher recoveries without deteriorating the analytical performance.

#### Effect of centrifugation

Generally, the centrifugation time hardly affects micelle formation, but it does accelerate phase separation in the same sense as conventional separation of a precipitate from its original aqueous environment. Centrifugation times of around 5–10 min are usually efficient for most CPE procedures [30,44]. The centrifugation time plays an important role in phase separation after formation of the cloud. A shorter centrifugation time is considered advantageous for CPE.

#### Effect of incubation and saturation

With increases in the temperature up to the cloud point, there is an increase in micellar size and a corresponding decrease in the CMC. The maximum analyte pre-concentration factor is reached when the CPE process is conducted with an equilibration temperature well above the cloud point temperature of the system. The use of elevated temperatures could result in decreased recovery because of decomposition. In the same way as other parameters, temperature and the duration of the CPE procedure seem to affect the extraction, especially when dealing with inert inorganic species. This reaction time coincides with the incubation time reported for the optimum extraction of organic species into micellar formations [18].

#### Effect of the Krafft temperature

The Krafft point of a surfactant is the temperature above which the solubility of the surfactant increases dramatically in an aqueous solution, and it is interpreted as the melting point of a hydrated solid surfactant [45]. The concept of the Krafft point has been applied extensively to ionic surfactants, but has rarely been observed for non-ionic surfactants. At the Krafft point, the solubility of the surfactant is equal to its CMC. Above the Krafft point, the total solubility of the surfactant increases dramatically because of micelle formation, resulting in efficient extraction [12].

### CPE coupled with microwave- or ultrasonic-assisted BE

CPE of non-ionic and anionic surfactants has been applied as a pre-concentration step before instrumental analysis. Because of the low volatility and high viscosity of the surfactant-rich phase, it cannot be injected directly for instrumental analyses such as high-performance liquid chromatography (HPLC), liquid chromatography-mass spectrometry, or gas chromatography (GC). Therefore, after CPE and before analysis, a supplemental stage is required to avoid injector blockages and column deterioration [32]. Microwave- or ultrasonicassisted BE is suitable for coupling CPE to instrumental analysis. A clean-up step is needed before injection for chromatographic analysis. In the BE procedure, the extracted surfactant-rich phase is treated with water or an immiscible solvent such as hexane, acetonitrile, ethyl acetate, iso-octane or chloroform. The target analytes are back extracted from the surfactant-rich phase to the organic solvent phase. In BE, the following three factors should be considered:

- *Effect of organic solvents:* among tested water-immiscible solvents for microwave-assisted BE, iso-octane was selected as the optimum solvent because it was the least volatile, and gave good reproducibility. The volume of iso-octane was optimized for good recovery of analytes from the surfactant-rich phase, a high pre-concentration factor, an appropriate volume for automated injection, and a relatively high analytical response [32]. In ultrasonic-assisted BE, hexane and ethyl acetate showed good results for pre-concentration of OP pesticides [2,32].
- *Effect of microwave irradiation or ultrasonication:* one of the most important tasks of this study was to evaluate the effects of microwave irradiation and ultrasonication on quantitative BE of pre-concentrated analytes from a surfactant-rich phase into an organic solvent. The time taken for BE depends on the microwave or ultrasonication power. Studies have shown that a microwave power of 700 W is effective for pre-concentration of OP pesticides (diazinon, quinalphos, fenthion, parathion-methyl and phorate) in urine [20], PAHs (naphthalene, acenaphthylene, fluorine, anthracene, fluoranthene and pyrene) in aqueous solutions [46], and diethylhexyladipate and acetyltributylcitrate in aqueous solutions [47]. Good pre-concentration of OP compounds (methidathion, chlorpyrifos, parathion and fenitrothion) in honey was achieved by ultrasonic-assisted BE under optimized conditions with 60 µL of hexane and an ultrasonication time of 20 min [32]. Dichlorovos, methamidophos, acephate, diazinon, dimethoate, chlorpyrifos, parathion-methyl, malathion and parathion-ethyl were effectively pre-concentrated from fruits using optimized BE with 200 µL of ethyl acetate and an ultrasonic time of 20 min [2].

## Reported literature

In any extraction technique, pre-concentration and separation are important for enhancing the analytical signals and lowering the limits of quantification and detection [48]. Several different compounds of forensic interest such as alkaloids, drugs of abuse and OP pesticides have been detected using various extraction techniques [49]. The literature presents a comprehensive account of recent CPE applications to alkaloids, drugs, and OP pesticides.

Qin et al. [17] developed and validated a CPE procedure using reversed-phase HPLC-with fluorescence detection for the determination and pre-concentration of the antidepressant drug venlafaxine in human plasma. Their proposed method had a high extraction efficiency (>90%), and a competitive analysis of the chromatograms obtained from plasma samples (blank, spiked and oral administration of the drug) showed that CPE successfully extracted the model analytes with no interference from the sample matrix or metabolic products of venlafaxine.

Abdollahi and Bagheri [50] utilized CPE for pre-concentration of a binary mixture of vitamin K_3_ and 1,4-napthoquinone. Both the model analytes were allowed to react with aniline in an initial phase, and this was followed by extraction under optimized conditions for aniline (0.033 mol/L), Triton X-114 (0.22% w/v), the equilibration time (15 min), and the cloud point temperature (25 °C). The study also showed the potential of the developed methodology for quantitative extraction and pre-concentration of intermediate reaction mixtures.

In a novel approach, Rukhadze et al. [33] developed an HPLC quantitative method for the determination of the free fractions of the anti-epileptic drugs carbamazepine and phenobarbital in complex biological matrices (e.g. plasma and saliva). In this study, CPE was used to remove the free and bound drug forms from plasma and serum, respectively. The CPE method gave high percentage recoveries (∼48% and 66%) for the carbamazepine and phenobarbital free fractions. This study also showed the superiority of CPE over conventional methodologies and highlighted several points of interest such as the ease of sample preparation, minimal sample requirements (∼200 μL of plasma), low cost, simple protocol and innate potential to concentrate varied analytes with enhanced pre-concentration factors. Consequently, it can be used for routine analysis, aid in envisaging the factors that influence drug-protein binding *in vivo* and *in vitro*, help with establishment of complex relationships among the total drug and free fractions and assessment of pharmacokinetics, and be used for direct determination of plasma- and serum-bound drugs (i.e. free and fractional forms).

Madej and Persona [51] developed an analytical methodology using reversed-phase HPLC—with diode array detection and CPE for the determination of six basic drugs (i.e. paracetamol, promazine, amitriptyline, nortriptyline, clomipramine and chlorpromazine) in human plasma. CPE gave efficient recoveries of drugs from complex biological matrices. The developed methodology was capable of detecting the model drugs at low (<22%) plasma concentrations. The proposed CPE methodology also exhibited good selectivity and specificity for slightly hydrophobic drugs such as paracetamol at very high concentrations.

Shen and Shao [16] pre-concentrated and simultaneously determined seven tobacco alkaloids (i.e. nicotine, nor-nicotine, myosmine, anabasine, nicotyrine, anatabine and 2,3-dipyridyl) from flue-cured leaves using CPE coupled with ultrasonic assisted back extraction (UABE). Screening of the extracted alkaloids was accomplished by gas chromatography-mass spectrometer (GC-MS). The outcomes of the study highlighted the advantages of this protocol, which required no pre-cleaning step before injection of the samples into the GC column for analysis. The developed CPE procedure resulted in efficient recoveries of the model analytes and the relative standard deviation range for the individual alkaloids was 2.77%–9.97% which is well within the prescribed range of International Conference on Harmonisation (ICH) guidelines [52].

Fontana et al. [32] proposed a coacervative CPE coupled with UABE (CCPE-UABE) for pre-concentration of OP pesticides from honey samples before quantification via GC-MS. The efficacy and potential applicability of the proposed CCPE-UABE-GC-MS method was tested by quantifying the amounts of OP pesticides in spiked and blank honey samples. The analysis showed that the proposed methodology had very high extraction efficiency (≥90%) and could extract methidathion at ultratrace levels (1.2–2.3 ng/g) from two honey samples.

Faria et al. [53] developed a CPE method for pre-concentration of the systemic insecticide and acaricide Disulfoton (Di-Syton) from water samples before quantification using GC. The developed methodology had very high extraction efficiencies of approximately 94% and 96% for the target from spiked water and river water samples, respectively. In contrast to liquid-liquid extraction procedures, the developed method showed enhanced efficiency, detectability, and reproducibility and was eco-friendly.

Tables 2–4 summarize the literature reports over the last few years on CPE applications to alkaloids, drugs and OP pesticides, respectively.

**Table 2.** Cloud point extraction (CPE) applications for the determination of alkaloids.

Alkaloids	Complex matrices	Surfactant system	Extraction optimized parameters						Detection method	Detection limit	Recovery (%)	Reference
			pH	Electrolyte	CPT (°C)	IT (min)	C (r/min)	CT (min)				
Nicotine, nornicotine, myosmine, anabasine, nicotyrine, anatabine, 2,3-dipyridyl	Tobacco leaves	5% (w/v) Triton X-114	9	NaCl	50	15	3 500	10	GC-MS	0.01 μg/mg	80.4	[16]
Indole: reserpiline alphayohimbine isoreserpiline 10-methoxy tetra-hydroalstonine	Leaves of *Rauwolfiate traphylla*	7% (w/v) Triton X-100	a	80 mol/L NaCl	50	30–60	4 000	20	HPTLC	0.20 μg/band 0.18 μg/band 0.30 μg/band 1.05 μg/band	95–97 98–116 96–116 93–99	[55]
Oleuropein hydroxytyrosol verbascoside luteolin-O-7-glucoside apigenin-O-7-glucoside	Olive leaf extract	4% (w/v) Tween 80	2.6	35% (w/v) Na_2_SO_4_	25	5	6 000	3	HPLC-DAD	b	99.8 93.0 99.3 87.6 100.0	[31]
Bergenin	*Ardisia japonica*	7% (v/v) Triton X-114	7	NaCl (3 g)	70	10	5 000	15	HPLC-UV	0.65 µg/mL	87.2	[18]
Δ^9^-tetrahydrocannabinol	*Cannabis* resin	1% (w/v) Dowfax 20B102	a	1% (w/v) Na_2_SO_4_	45	30	4 000	10	HPLC-UV	0.04 μg/mL	81	[56]
Flavonoids: rutin hyperoside quercetin-3-O-sophoroside isoquercitrin astragalin quercetin	*Apocynumvenetum* leaf sample	1.2% (w/v) Genapol X-080 solution and 0.1% (w/v) CTAB	8.0	1.0% (w/v) NaCl	55	10	4 000	5	HPLC-PAD	20.0 ng/mL 5.0 ng/mL 5.0 ng/mL 10.0 ng/mL 10.0 ng/mL 10.0 ng/mL	95.1 96.2 96.9 95.4 94.4 94.8	[37]
Natural antioxidants (phenols)	Olive mill wastewater	4%–6% (w/v) Triton X-114	b	b	55–66	20	3 500	5	UV-visible spectro- photometer	b	>96	[57]
Aesculin aesculetin	*Cortex fraxini*	5% (w/v) Genapol X-080	1	20% (w/v) NaCl	55	30	b	b	HPLC-UV	b	60.29 41.47	[36]
Daidzein	*Puerariae radix*	5% (w/v) Genapol X-080	a	NaCl	50	10	a	a	HPLC-UV	0.07 µg/mL	90.9–106.7	[35]
Anthraquinones	*Morindacitrifolia*	1% (w/v) Triton X-100	a	NaCl	75	30	4 000	10	HPLC-UV	b	95	[58]

CPT: cloud point temperature; IT: incubation time; C: centrifugation; CT: centrifugation time; GC-MS: gas chromatography-mass spectrometer; HPTLC: high-performance thin layer chromatography; HPLC-DAD: high-performance liquid chromatograph with diode array detector; HPLC-UV: high-performance liquid chromatograph with ultraviolet detector; HPLC-PAD: high-performance liquid chromatography with pulsed amperometric detector; UV: ultraviolet detector. a: not required; b: data not provided.

**Table 3.** Cloud point extraction (CPE) applications for the determination of drugs.

Drugs	Complex matrices	Surfactant system	Extraction optimized parameters						Detection method	Detection limit	Recovery (%)	Reference
			pH	Electrolyte	CPT (°C)	IT (min)	C (r/min)	CT (min)				
Arbidol	Rat plasma	5% (w/v) Triton X-114	a	1 mol/L NaCl	45	20	3 500	5	HPLC-UV	0.08 µg/mL	>89.7	[59]
Osthole	Rat plasma	0.8% (w/v) Triton X-114	a	0.4 mol/L NaCl	45	20	3 500	5	HPLC-UV/PDA	0.03 μg/mL	90.4	[60]
Carbamazepine phenobarbital	Human blood plasma and saliva	5% (w/v) Triton X-114	7	a	35	10	3 000	5	HPLC-UV	0.6–0.7 µg/mL	b	[33]
Phenothiazines: pericyazine chlorpromazine fluphenazine	Human serum	7.5% (w/v) Triton X-114	a	a	60	10	a	a	GC-FID	a	95.1 87.1 84.7	[61]
Venlafaxine	Human plasma	5% (w/v) Triton X-114	a	0.3 mol/L NaCl	40	20	5 000	5	HPLC-FD	2 ng/mL	89–93	[17]
Paracetamol	Human urine	2% (w/v) Triton X-114	a	a	25	a	a	a	UV-visible	a	100	[61]
Salicylic acid	Human urine	1% (w/v) Triton X-114	a	a	40	10	a	a	UV-visible	a	85–94	[61]
Flurbiprofen	Rat plasma	5% (w/v) Genapol X-080	a	a	50	20	3 500	5	HPLC-UV	0.1 µg/mL (LLOQ)	>84.5	[19]
Paracetamol promazine amitriptyline chlorpromazine salicyclic acid opipramol carbamazepine alprazolam	Human plasma/serum	7.5% (w/v) Triton X-114	12 bd 6 ad/nd	a	40	30	13 000	10	HPLC-DAD	– 0.125 µg/mL 0.25 µg/mL 0.5 µg/mL – 1 µg/mL – –	25.2 107.9 81.5 83.8 62.7 25.7 55.5 27.9	[62]
Basic drugs: paracetamol (P) promazine amitriptyline nortriptyline clomipramine chlorpromazine	Human plasma	7.5% (w/v) Triton X-114	12	a	25	20	13 000	10	RP-HPLC/DAD	0.5 µg/mL 0.12 µg/mL 0.25 µg/mL 0.25 µg/mL 0.5 µg/mL 0.12 µg/mL	22.08 94.11 103.6 91.98 86.28 82.49	[51]
Phthalate: diethylphthalate di-(2-ethylhexyl)-phthalate di-cyclohexyl-phthalate	Environmental water samples	0.25% (w/v) Triton X-114	–	0.4 mol/L Na_2_SO_4_	45	60	3 500	5	HPLC-UV	2.0 ng/ml 3.8 ng/ml 1.0 ng/ml	98.7 86.5 98.8	[38]
Terazosin hydrochloride (THD): 2-[4-(2-tetrahydrofura-nyl carbonyl]-1-piperazinyl-6,7-dimethoxy-4-quinazolinamine monohydro-chloride dehydrate	Human urine	0.25% (w/v) PONPE 7.5	10	0.002 mol/mL NaClO_4_	a	a	3 500	10	UV-visible spectro-photometer	0.0003 µg/mL	98	[28]
Estrogens: estriol estradiol estrone progesterone	Water	0.25% (w/v) Triton X-114	a	0.4 mol/L Na_2_SO_4_	40	60	3 500	5	HPLC–UV	0.23 ng/mL 0.32 ng/mL 0.25 ng/mL 5.0 ng/mL	99 81.2 98 92.1	[40]
Vitamin A vitamin E	Human serum and whole blood	Genapol X-80	a	0.125 NaCl	40–60	10	3 500	5	RP-HPLC-UV	b	85.6 60.4	[63]
Vitamin K_3_ 1,4-naphthoquinone	Real and synthetic mixture	0.22% (w/v) Triton X-114	a	a	25	15	3 500	5	UV-visible spectro-photometer	0.05 µg/mL 0.08 µg/mL	85 80	[50]
Ciprofloxacin	Blood serum	1% (w/v) Triton X-114	a	Fe (III)	75	25	6 000	20	UV-visible spectro-photometer	0.77 µg/mL	98.89	[64]
Penicillin: ampicillin penicillin G oxacillin cloxacillin	Bovine milk	1.5% (w/v) Triton X-114 0.06% (w/v) CTAB	8	7% (w/v) Na_2_SO_4_	40	5	3 000	20	HPLC-UV	2 ng/mL 2 ng/mL 2 ng/mL 3 ng/mL	81.9 95.5 95.4 95.5	[65]
Xanthohumol	Beer	2.5% (v/v) Triton X-114	5	15% (w/v) NaCl	70	10	a	a	HPLC-UV	0.003 mg/L	90.7–101.9	[66]

CPT: cloud point temperature; IT: incubation time; C: centrifugation; CT: centrifugation time; HPLC-UV: high-performance liquid chromatograph with ultraviolet detector; HPLC-PAD: high-performance liquid chromatography with pulsed amperometric detector; GC-FID: gas chromatograph(y)-flame ionization detector; HPLC-FD: high-performance liquid chromatography with fluorescence detection; UV: ultraviolet detector; bd: basic drug; ad/nd: acidic drug/neutral drug; RP-HPLC-UV: reverse phase high-performance liquid chromatography with ultraviolet detector; LLOQ: lower limit of quantification. a: not required; b: data not provided.

**Table 4.** Cloud point extraction (CPE) applications for the determination of organophosphorus (OP) pesticides.

OP pesticides	Complex matrices	Surfactant system	Extraction optimized parameters						Detection method	Detection limit	Recovery (%)	Reference
			pH	Electrolyte	CPT (°C)	IT (min)	C (r/min)	CT (min)				
OPs: dichlorvos methamidophos acephate diazinon dimethoate chlorpyrifos parathion-methyl malathion parathion-ethyl	Fruit juice	6% (w/v) polyethylene glycol 6000 (PE 6000)	6	20% (w/v) Na_2_SO_4_	a	a	4 000	5	GC-FPD	1.5 μg/kg	71.6	[2]
										2.0 μg/kg	77.4	
										3.0 μg/kg	72.0	
										0.5 μg/kg	93.6	
										2.0 μg/kg	76.4	
										1.0 μg/kg	81.4	
										1.0 μg/kg	89.2	
										1.0 μg/kg	94.6	
										1.5 μg/kg	93.0	
Triazine: simazine cyanazine simetryn atrazine	Milk	Triton X-100	6	0.9% Na_2_SO_4_	60	30	9 917	10	HPLC-UV	6.79 µg/L	86.4	[42]
										11.19 µg/L	92.3	
										7.98 µg/L	96.9	
										10.95 µg/L	75.2	
Carbofuran (2,3-dihydro-2,2-dimethyl-7-benzofuranyl meth-ylcarbamate)	Rice sample	4% (w/v) Triton X-100	9.5	18% (w/v) Na_2_SO_4_	30	10	a	a	HPLC-UV	0.0005 mg/kg	87.9–91.7	[67]
OPs: phorate diazinon parathion-methyl fenthion quinalphos	Human urine	1.4 mol/LTriton X-114	6	1 ng/mL NaCl	50	15	4 000	10	GC-FPD	0.07 ng/mL	76.9	[20]
										0.04 ng/mL	88.2	
										0.08 ng/mL	70.9	
										0.07 ng/mL	72.8	
										0.07 ng/mL	73.4	
OPs: dimethoate methidathion parathion-methyl malathion ethoprophos parathion-ethyl diazinon chlorpyrifos	Wastewater sample	4% (w/v) Genapol X-080	a	4% and 3% NaCl	90	15	a	a	LC-UV	0.61 ng/mL	29	[68]
										1.37 ng/mL	85	
										0.03 ng/mL	83	
										1.37 ng/mL	81	
										1.84 ng/mL	47	
										0.36 ng/mL	100	
										0.47 ng/mL	84	
										2.81 ng/mL	99	
OPs: chlorpirifos fenitrothion parathion methidathion	Honey	100% (w/v) Triton X-114	2	a	85	5	3 500	5	GC-MS	0.06 ng/mL	95	[32]
										0.03 ng/mL	100	
										0.09 ng/mL	90	
										0.47 ng/mL	90	
Disulfoton	Surface water	1.0% (w/v) Triton X-114	b	0.5 mol/L NaCl	40	10	3 500	10	GC	1.20 µg/L	∼100	[53]
OPs: paraoxon methyl-parathion fenitrothion ethyl-parathion	Water sample	1.0% (w/v) Triton X-114	b		40	5	3 500	10	LC	0.35 ppb	80–100	[4]
										0.21 ppb		
										0.18 ppb		
										0.33 ppb		

CPT: cloud point temperature; IT: incubation time; C: centrifugation; CT: centrifugation time; GC-FPD: gas chromatograph with flame photometric detector; HPLC-UV: high-performance liquid chromatograph with ultraviolet detector; LC-UV: liquid chromatograph with ultraviolet detector; GC-MS: gas chromatography-mass spectrometer; GC: gas chromatograph; LC: liquid chromatograph. a: not required; b: data not provided.

### Analytical applications

Methods for analysis of alkaloids, organic dyes, drugs and OP pesticides have been established using ultraviolet-visible spectrophotometry, spectroflorometry, HPLC and GC [54]. However, in some situations, the sample needs separation and pre-concentration steps before analytical measurement. CPE has been used for the extraction and pre-concentration of many analytes, such as drugs, pesticides, alkaloids and metal ions. Table 1 summarizes the available literature from the last few years on applications of CPE to analysis of alkaloids, drugs, and OP pesticides. It includes the type of analyte, matrix, micellar system, optimized parameters for the extraction, detection method, detection limit and recovery data.

## Forensic prospects

Many extraction methods are used in forensic science laboratories to extract analytes from complex matrices such as blood, urine, viscera, hair, soil, saliva and milk into organic solvents. Surfactant-based extraction is simple, reliable and compatible with micellar chromatography or micellar electrokinetic chromatography. Non-ionic surfactants such as Triton X-114 [69], Triton X-45, Triton X-100, Dowfax 20B102 [56], polyoxyethylene (5.0) nonylphenol (PONPE 5.0) [70], and PONPE 5 are good for extraction because the micellar system containing the complex was thermostated at 30 °C in order to promote phase separation. Tergitol 15-S-9, Neodol 25-7, Tergitol 15-S-7 [71], Brij-35, and Brij-97 [72] have been used as extractants for metals ions, alkaloids, pesticides, drugs, dyes, PAHs and other analytes. CPE coupled with BE can be scaled-up in forensic toxicology and chemical analysis via modification of the surfactant solutions according to the extraction target to exploit the research area and meet green chemistry principles. Several aqueous surfactant solutions have been used instead of organic solvents in classical extraction protocols. It is important to compare classical extraction methods with CPE and to investigate the application of CPE to solid samples (e.g. postmortem human tissues and viscera), which are frequently encountered in forensic chemical and toxicological analyses. Studies should evaluate the extraction time, solvent, and reproducibility in CPE for solid biological samples. Eszopiclone can be extracted from complex matrices and mouse blood *in vivo* using CPE-BE [73]. This area of analysis is especially attractive for the development of CPE methodologies. Hair, viscera, teeth, saliva, nails, and other solid materials are frequently encountered in forensic investigations, and the sample preparation and extraction methods can be time-consuming [74,75]. CPE may shorten and simplify the analysis, especially for hair samples. This is a promising method for development of effective techniques for extraction of analytes from various complex matrices.

## Conclusion

This review shows that CPE-BE is an analytical tool that has great potential for improving detection limits and other analytical characteristics. It is a valid alternative to separation and pre-concentration procedures because of its high recoveries and concentration factors. In addition, the surfactants used in CPE-BE make the micellar-based extraction procedure simple, practical, safe and economical. Micellar extraction is a promising field for the development of new and effective analytical methods for different matrices. Surfactant assemblies/solutions and surfactant-mediated CPE should be improved because of their important roles in the extraction of various analytes. This method could be scaled-up for forensic science, toxicology and pharmaceutical applications.
